# Antioxidant and Fatty Acid Changes in Pomegranate Peel With Induced Chilling Injury and Browning by Ethylene During Long Storage Times

**DOI:** 10.3389/fpls.2022.771094

**Published:** 2022-03-09

**Authors:** Mónika Valdenegro, Lida Fuentes, Maricarmen Bernales, Camila Huidobro, Liliam Monsalve, Ignacia Hernández, Maximiliano Schelle, Ricardo Simpson

**Affiliations:** ^1^Escuela de Agronomía, Pontificia Universidad Católica de Valparaíso, Quillota, Chile; ^2^Centro Regional de Estudios en Alimentos Saludables (CREAS), CONICYT-Regional GORE Valparaíso Proyecto R17A10001, Valparaíso, Chile; ^3^Instituto de Química, Bioquímica, Pontificia Universidad Católica de Valparaíso, Valparaíso, Chile; ^4^Departamento de Ingeniería Química y Ambiental, Universidad Técnica Federico Santa María, Valparaíso, Chile

**Keywords:** chilling injury, postharvest pomegranate, cold storage, total polyphenols, anthocyanins, fatty acids

## Abstract

Pomegranate (*Punica granatum*) is a non-climacteric fruit with a high antioxidant content in arils and peels, of which 92% are anthocyanins and tannins. However, it is susceptible to chilling injury (CI), a physiological disorder concentrated in the peel, which can affect the organoleptic quality of the fruit. To understand the effects of modified atmosphere and ethylene in responses to stress on the antioxidant quality of the fruit and composition of fatty acids in the peel under CI conditions, the exogenous ethylene treatments (0.5, 1.0, and 1.5 μg L^–1^), 1-methylcyclopropene (1-MCP; 1 μl L^–1^), modified atmosphere packaging (MAP: XTend™ bags), combined strategy MAP/1-MCP, and package in macroperforated bags (MPB-control treatment) were evaluated. The assay was performed in cold conditions (2 ± 1°C; 85% RH) to stimulate damage and was sampled for 120 days (+3 days at 20°C). During cold storage, CI symptoms began at 20 days in MPB and at 60 days for all treatments with exogenous ethylene; CI symptoms were delayed up to 120 days in MAP, 1-MCP, and the combined MAP/1-MCP treatment. Damage was concentrated in the peel. Ethylene and MPB-control treatments induced significant electrolyte leakage, lipid peroxidation, and oxidative damage. In contrast, MAP alone or in combination with 1-MCP successfully delayed CI symptoms. However, no significant differences were observed between treatments in fatty acid content, e.g., in the peel, oleic acid, linoleic acid, palmitic acid, but a significant loss was noted after 60 days of storage. Cold storage caused an increase in anthocyanin concentration in the peel and arils, increasing up to 12 times in the peel of the fruit treated with ethylene at the final stage of storage (120 days + 3 days at 20°C), with non-significant differences in the tannin content in the peel. During long-term cold storage of pomegranate, MAP and 1-MCP treatments delay and reduce the appearance of CI symptoms. This long cold storage induces an important decrease in the unsaturated/saturated fatty acid ratio, which is not reversed by any postharvest treatment. A higher unsaturated/saturated fatty acid ratio after 1-MCP treatments showed a protective effect in peel tissues. In addition, it was possible to increase the concentration of anthocyanins in the peel of cold-storage pomegranates treated with ethylene.

## Introduction

Pomegranate (*Punica granatum*) is a non-climacteric fruit (Kader, 2003) belonging to the family *Punicaceae* (Selcuk and Erkan, 2015) that grows in tropical and subtropical regions. Pomegranate fruit has bioactive molecules such as polyphenols, tannins, anthocyanins, principally ellagitannins, and phenolic acids, which give it health properties (Pirzadeh et al., 2020). However, the short harvest period and long travel times induce significant quality loss, which reduces the storability and affects the consumer acceptance of these fruits (Fawole and Opara, 2013; Matityahu et al., 2013; Pareek et al., 2015; Selcuk and Erkan, 2015). Therefore, within the changes associated with the loss of quality, one can observe shriveling, decay, weight loss, development of superficial scald, chilling injury (CI), and a decrease in flavor acceptability (Gamrasni et al., 2015; Li et al., 2016).

One of the most critical problems during storage is CI, a complex physiological disorder that affects the appearance and organoleptic attributes of the fruit (Sala, 1998). This physiological disorder is mainly observed in fruits and vegetables when storage is below the threshold temperatures for too long a time, resulting in metabolic dysfunction and finally noticeable chilling symptoms (Farneti et al., 2015). In pomegranate, the CI has several undesirable effects on the quality that can be more visible, such as surface pitting, brown discoloration of the peel, husk scald, reduced color and hardness, and susceptibility to fungal development. Additionally, these changes can include internal damage or more subtle changes, such as aroma degradation, arils of pale color, and white separating segments of the arils with brown discoloration from exposure at temperatures below 5°C during 2 months (Artés et al., 2000; Fawole and Opara, 2013).

One of the principal characteristics of CI is the change in the structure and composition of the membrane, with the consequent loss of permeability regulation and metabolic disorders (Sala, 1998; Ben-Amor et al., 1999; Li et al., 2016). It has been suggested that a higher ratio of unsaturated and saturated fatty acids (SFAs) provides major tolerance to cold damage by chilling temperature in fruits such as banana, loquat, mango, and peach, preventing ion leakage, membrane peroxidation, and final tissue damage (Promyou et al., 2008; Jin et al., 2014; Li et al., 2014).

The browning of peel pomegranate is an essential problem at these cold temperatures and can be associated with the accumulation of brown polymeric pigments by enzymatic activity. It has been suggested that tannins are the basic substance of pomegranate peel browning (Zhang et al., 2008). Furthermore, the browning index of the peel has been positively correlated with polyphenol oxidase (PPO), the enzyme responsible for phenolic compound oxidation into quinone compounds, and negatively correlated with catalase (CAT) activity (Zhang et al., 2008). Therefore, an oxidative stress phenomenon associated with decreases in the enzymatic reactive oxygen-scavenging mechanism, i.e., CAT, peroxidase (POX), and superoxide dismutase (SOD) activity has been suggested during cold storage of pomegranate (Pech et al., 2008).

In recent years, the effect of different treatments on reducing CI during cold storage of pomegranate has been studied. Methyl jasmonate and acetylsalicylic acid reduce CI symptoms by decreasing respiration, softening, and ion leakage (Sayyari et al., 2011, 2017; Chen et al., 2021). Furthermore, oxalic acid’s reduction in CI symptoms is related to lower losses of total phenolics and increased ascorbic acid content in arils (Sayyari et al., 2011). However, salicyloyl chitosan treatment has been associated with alleviating CI by maintaining membrane integrity resulting from a higher unSFA/SFA ratio and enhancing antioxidant capacity by total phenols, anthocyanins, and ascorbic acid accumulation (Sayyari et al., 2016). The postharvest strategies related to reducing ethylene production and the respiratory rate of the pomegranate can have a positive impact on the quality and consumer acceptance of pomegranate after long cold storage. For example, a modified atmosphere is an effective strategy in reducing CI during the storage of mango fruits at 12°C (Pesis et al., 2000), and treatment with ethylene inhibitor 1-methylcyclopropene (1-MCP) improves the postharvest storage of pomegranate (Defilippi et al., 2006; Gamrasni et al., 2015; Li et al., 2016). Previously, it was reported that endogenous ethylene biosynthesis induced by ethrel application accelerated the appearance of CI symptoms, in contrast to observations made for modified atmosphere and 1-MCP treatments during the long-term cold storage of pomegranate (Valdenegro et al., 2018). Additionally, ethrel at 1 or 1.5 μg L^–1^ accelerates endogenous ethylene production at 20 days relative to cold-induced ethylene production at 160 days (Valdenegro et al., 2018). However, the mechanisms by which ethylene increases or accelerates CI in pomegranate are unknown. Therefore, this study aimed to evaluate the relationship among antioxidant response, fatty acid composition, and CI under ethrel and 1-MCP applications during cold storage at 2°C of the pomegranate fruit.

## Materials and Methods

### Plant Material

The pomegranate (*Punica granatum*) cv. Wonderful fruits were collected from 6-year-old trees from an orchard in Curacaví (–33.318S, –71.123W, 224 m.a.s.l.), Chile. The drip irrigation system and pomegranate tree culture were made at approximately 5,388 m^3^ ha^–1^ year^–1^ (Holland et al., 2009; Franck et al., 2015), and the fertilization used was 350, 165, and 85 kg ha^–1^ year^–1^ for potassium, nitrogen, and phosphorus, respectively (Franck et al., 2015).

The fruits were harvested and selected on the basis of maturity, considering size, weight, color, and other quality parameters according to Valdenegro et al. (2018), before experimental treatments.

### Experimental Strategy

The pomegranates were randomly divided into eight groups of 180 fruits as described in Valdenegro et al. (2018). The experimental unit was formed by ten fruits, selected by sanity and size, in the mature stage (16°Brix). The treatments were performed as follows (details in Table 1): The first group consisted of packing in macroperforated bags (MPB-control) like control groups without treatment or modification of atmosphere around the fruit, according to previous studies of the modified atmosphere (Hussein et al., 2015). In the second group, ten fruits per bag were packed in commercial conditions under a passive modified atmosphere (MAP: commercial bag MAP XTend™ film (StePac, São Paulo, Brazil), according to details described in Valdenegro et al. (2018). Bag-sealed films were dimensioned as follows: 80 × 56 cm^2^ with a 0.002% perforated area, according to previous studies (Porat et al., 2009). The third group was treated with 1-MCP (SmartFresh™) according to the specifications of the manufacturer (1 μg L^–1^) and Valdenegro et al. (2018). The fourth group was subjected to combined treatment with MAP and 1-MCP. Finally, the three treatments at different levels of increasing exogenous application of analogous ethylene (E), E1, E2, and E3, were performed by dipping the fruit in 0.5, 1, or 1.5 μg L^–1^ Ethrel™ solution for 3 min followed by drying (Valdenegro et al., 2012). After all these treatments, the fruits were stored at 2 ± 1°C to stimulate chilling symptoms and sampled at 0, 20, 60, and 120 days, followed by storage for 3 days at 20°C to simulate shelf life conditions. Each sample unit was composed of ten fruits per treatment and the sampling date was evaluated in triplicate. The quality parameters (color, acidity, and total soluble solids) and physiological parameters were evaluated as described by Valdenegro et al. (2018). After fruit quality assessments, the peel and arils were separated for further analysis. The fruit peel was cut into small pieces, and both tissues were frozen under liquid nitrogen and stored at −80°C until fatty acid profile and antioxidant and non-antioxidant enzymatic systems evaluation.

**TABLE 1:** Treatment tested for the study of chilling injury in pomegranate fruit.

Treatment	Details
Macroperforated bags control (MPB-control)	No treatment, stored in air
Passive modified atmosphere (MAP)	Commercial bag MAP XTend™ film (StePac, São Paulo, Brazil), stored in the air
1-MCP	1-MCP (SmartFresh™), 1 μg L^–1^, stored in air
Combined MAP/1-MCP	Combinated MAP/1-MCP, stored in air
E1	0.5 μg L^–1^ Ethrel™, dipping 3 min, stored in air
E2	Ethrel™ (1.0 μg L^–1)^, dipping 3 min, stored in air
E3	Ethrel™ (1.5 μg L^–1)^, dipping 3 min, stored in air

### Determination of Chilling Injury Indicators

The visual estimation of CI was determined in peel and aril tissues by rating the extent of surface pitting and browning using a grading scale ranging from non-symptomatic (0) to 4 (severe symptoms 100%) according to Sayyari et al. (2009) as described by Valdenegro et al. (2018). Additionally, the symptom severity was evaluated according to the percent (0–100%) of damage in the fruit surfaces.

### Determination of Membrane Damage: Peel Electrolyte Leakage and Lipid Peroxidation by Malondialdehyde Determination

The cell membrane damage in pomegranate peels was measured as the percentage of electrolyte leakage according to Ben-Amor et al. (1999) and modified by Valdenegro et al. (2018). Briefly, ten disks of 1 cm in diameter were cut from different areas of the peel of each sample. The samples were incubated in 40 ml of 0.4 mol L^–1^ mannitol for 2 h at 25°C. Then, the samples were frozen for 12 h, autoclaved at 120°C for 35 min, and finally cooled at ambient temperature (20°C). The percentage of electrolyte leakage was calculated as the ratio of initial to total conductivity of the samples.

The level of lipid peroxidation in cell membranes was measured using 2-thiobarbituric acid-reactive substances, mainly malondialdehyde (MDA), following the method of Vendruscolo et al. (2007), with the modification described in Valdenegro et al. (2018). Ten grams of peel sample was ground in liquid nitrogen and extracted in 40 ml of 10% trichloroacetic acid. A 2-ml aliquot of the supernatant obtained by centrifugation at 8,000 × *g* for 10 min was mixed with 2 ml of 10% trichloroacetic acid containing 0.6% thiobarbituric acid. The mixture was heated at 95°C for 30 min and then quickly cooled in an ice bath. After the tube was centrifuged at 8,000 × *g* for 10 min, the absorbance at 532 nm was read. The MDA concentration was calculated using its molar extinction coefficient (155 mM cm^–1^). The results are expressed as μmol of MDA per g of fresh weight (FW).

### Determination of Fatty Acid Methyl Ester Methods

The pomegranate oil was extracted according to Hernández et al. (2017). The fatty acids in the pomegranate peel oil were determined using the method described by Chirinos et al. (2013), with modifications. The pomegranate fatty acid methyl esters (FAMEs) were analyzed using external standards in an Agilent 7890B gas chromatograph equipped with a flame ionization detector (GC-FID) with a phenylalanine ammonia-lyase (PAL)3 autosampler (Agilent Technologies, Santa Clara, CA, United States) and an SPTM-2560 fused silica capillary (100 m × 0.25 mm × 0.2 μm) column (Sigma Chemical Co., St. Louis, MO, United States). One microliter was injected in split mode (1:50) at 220°C. The oven temperature was programmed to start at 80°C, increase to 175°C at 25°C min^–1^, hold at 175°C for 25 min, increase to 205°C at 10°C min^–1^, hold at 205°C for 4 min, increase to 225°C at 10°C min^–1^, hold at 225°C for 20 min, and finally, decrease to 80°C at 20°C min^–1^. The detector temperature was set at 225°C. Helium (Indura, Santiago, Chile) was used as a carrier gas at a constant flow of 1.6 ml min^–1^. The results were expressed as mg of fatty acid per g of pomegranate dried weight (DW).

### Browning in Peel and Aril Tissues: Phenylalanine Ammonia-Lyase (EC 4.3.1.24) and Polyphenol Oxidase (EC 1.10.3.2) Activity Determination

The *phenylalanine ammonia-lyase activity* was measured on the basis of the conversion of L-phenylalanine to *trans*-cinnamic acid, according to the method described by Peltonen and Karjalainen (1995). Briefly, 50 mg of polyvinylpyrrolidone (PVP) and 4 ml of 0.1 M Tris–HCl buffer (pH 8.9) including 10 mM β-mercaptoethanol were added to 500 mg of ground pomegranate tissue, peel, and arils. The extracts were centrifuged at 10,000 × *g* for 20 min at 4°C. Five hundred microliters of tissue extract, 1,000 μl of 80 mM borate buffer (pH 8.9), and 30 mM phenylalanine were mixed and incubated for 1 h in a water bath at 30°C, and then 1.5 ml of 2 M HCl was added. Finally, the concentration of trans-cinnamic acid was determined at 290 nm, and the PAL activity was expressed as nmol of cinnamic acid/min mg of protein.

The *polyphenol oxidase activity* was evaluated by UV/Vis spectrophotometry using Agilent 845X equipment (Santiago, Chile) according to the methodology set forth by Espin et al. (1995). For this, the crude extract was prepared, homogenizing 50 g of pomegranate peel with 100 ml of 0.2 M sodium phosphate buffer at pH = 7 in an ultra-Turrax T-18 (IKA, Scaufen, Germany). The homogenate was centrifuged at 18,000 × *g* at 4°C for 30 min in a Solvall RC-5B centrifuge (Germany), and the supernatant was separated with a Whatman #1 filter (Whatman, Great Britain). The enzyme extract was kept frozen at −80°C until analysis. The PPO activity is expressed as delta activity/min/mg of protein.

### Enzymatic Antioxidant Activity in Peel Tissue: Catalase (EC.1.11.1.6), Guaiacol Peroxidase (EC.1.11.1.7), and Superoxide Dismutase (EC.1.15.1.1)

The *catalase* (EC.1.11.1.6) activity was determined according to Aebi (1984). Then, 1.5 ml of 100 mM sodium phosphate buffer (pH 7.0) containing 2% polyvinyl pyrrolidone (PVPP) and 1.4 mM of ethylenediaminetetraacetic acid (EDTA) was added to 350 mg of ground peel. After centrifugation at 20,000 *g* for 15 min at 4°C, the supernatant was used for the enzymatic assay. The reaction mixture contained 30 mM H_2_O_2_ in 50 mM phosphate buffer (pH 7.0) and 100 μl peel extract in a total volume of 1 ml. The CAT activity was estimated by a decrease in absorbance of H_2_O_2_ at 240 nm and was expressed as units/mg protein.

The *guaiacol peroxidase activity* (EC.1.11.1.7) was determined as the oxidation of guaiacol by the rate of tetraguaiacol formation at 470 nm (ε = 26,600 mM^–1^ cm^–1^) (Rao et al., 1998). The assay mixture (195 μl) contained 16 mM guaiacol, 16.2 μl 88 mM H_2_O_2_, 60 μl 500 mM potassium phosphate buffer (pH 7.0), and 2.5 μl 10 mM EDTA (pH 7.0). The reaction was initiated by adding 105 μl of peel protein extract. The results were expressed as Unit/mg protein.

The *superoxide dismutase activity* (EC.1.15.1.1) was determined through a spectrophotometric assay based on the reduction of highly water-soluble tetrazolium salts by xanthine-xanthine oxidase (Ukeda et al., 1999). The results were expressed as Unit/mg protein. The protein content was measured for all enzymatic activities according to Bradford (1976).

### Non-enzymatic Antioxidant Activity: Trolox Equivalent Antioxidant Capacity and (1,1-diphenyl-2-picrylhydrazyl) free radical (DPPH)

The antioxidant activity by (1,1-diphenyl-2-picrylhydrazyl) free radical (DPPH) assay (Brand-Williams et al., 1995) was determined using Trolox as the standard. Briefly, 100 μl of methanol extract dilution of peel was mixed with 3.9 ml of 103.5 μM solution of DPPH radical in methanol and incubated at room temperature for 60 min in the dark. The reduction of the DPPH radical was measured at 517 nm in a UV/Vis spectrophotometer (model Lambda 25, Perkin Elmer). The standard curve was generated with 0.1–0.7 mM 6-hydroxy-2,5,7,8-tetramethylchroman-2-carboxylic acid (Trolox, #238813, Sigma Chemicals) in methanol. The results are expressed as% inhibition and IC_50_ (μg/ml).

The Trolox equivalent antioxidant capacity (TEAC) assay (van den Berg et al., 1999) was performed with modifications (Valdenegro et al., 2012) using Trolox as the standard. The methanol extract from peel was diluted in phosphate-buffered saline (PBS) solution (pH 7.4), and 40 μl was mixed with 1.96 ml of the radical solution. The decrease in the optical density (OD) at 734 nm in a UV/Vis spectrophotometer (model Lambda 25, Perkin Elmer) was recorded for 6 min. The results were expressed as mg Trolox equivalents (TE)/g FW.

### Polyphenol, Anthocyanin, and Hydrolyzable Tannin Contents

The total polyphenols were determined according to Folin- Ciocalteu’s method. Forty milliliters of methanol/HCl (99:1 v/v) was added to 9 g frozen tissue (peel or arils). Each sample was homogenized for 10 min using an ultra-Turrax T-18 (IKA, Scaufen, Germany) and centrifuged for 20 min at 16,000 × *g*. The supernatant solution was filtered using 0.22 μm cellulose filters. The extraction procedure was repeated two times for each sample. The spectrophotometric measurements were carried out using a UV/Vis spectrophotometer (model Lambda 25, Perkin Elmer). The sample extract (5 ml) was introduced in a 20 ml volumetric flask; 5 ml of Folin Ciocalteu’s reagent and 10 ml of saturated sodium carbonate solution (75 g/L) were added and mixed. The solution was brought to 100 ml with water. After incubation at room temperature for 2 h, the absorption at 750 nm was measured. The results were expressed as mg of gallic acid per 100 g of fresh tissue.

The hydrolyzable tannins were determined by the method of Bossu et al. (2006) with slight modifications. Ground aril or peel tissue (0.25 g) was extracted with 50 ml of methanol (80%) with agitation at room temperature for 6 h. After centrifugation (10 min, 2,000 rpm), the fruit extract was diluted three times with water; then, 1 ml of the diluted sample was added to 5 ml of KIO_3_ aqueous solution (2.5% w/v) tempered at 30°C, and the absorbance was measured at 550 nm. The standard curve was obtained using tannic acid solution (5,000 mg/L). The results were expressed as mg tannic acid equivalent (TAE) per g of dry weight (DW).

The total anthocyanins were calculated as cyanidin-3-glucoside and were determined by means of the pH differential method (Giusti and Wrolstad, 2001) using two buffer systems: potassium chloride buffer, pH 1.0 (0.025 M), and sodium acetate buffer, pH 4.5 (0.4 M). An aliquot of the fruit extract anthocyanin solution was adjusted to pH 1.0, and another aliquot was adjusted to pH 4.5. Each 0.2 ml aliquot (two replicates) of the fruit extract was diluted with 1.8 ml of pH 1.0 buffer or with pH 4.5 buffer. Each solution was allowed to stand at room temperature for 20 min. The absorption was measured using a UV/Vis spectrophotometer (model Lambda 25, Perkin Elmer) at 510 and 700 nm.

The total anthocyanin content was calculated based on the Lambert–Beer law using the coefficient of molar extinction for cyanidin-3-glucoside (26,900 M^–1^ cm^–1^). The results of four extractions from each treatment at each postharvest time point were expressed as mg of cyanidin-3-glucoside equivalent per 100 g of FW.

### Sensory Determinations

Ten trained panelists evaluated the pomegranate sensory quality for sweetness, color and luminosity, aroma, and sourness after 20, 60, and 120 days of storage at 2°C (+3 days at 20°C). The evaluation was performed on a scale of 1–10 (1 = weakest and 10 = excellent). The average of scores was used for evaluation (Ehteshami et al., 2020).

### Statistical Analysis

The experimental design was two-factorial, and the sources of variation were postharvest treatment and the date of sampling. Statistical analyses were performed using SPSS v.14. A two-way ANOVA was performed. Mean comparisons were performed using the Honestly Significant-Difference (HSD) test at *P* ≤ 0.05. The results of the chemical analysis are presented as the mean values ± SE considering five independent extractions and five technical replicates.

## Results and Discussion

### Average Chilling Injury Index

**FIGURE 1:**
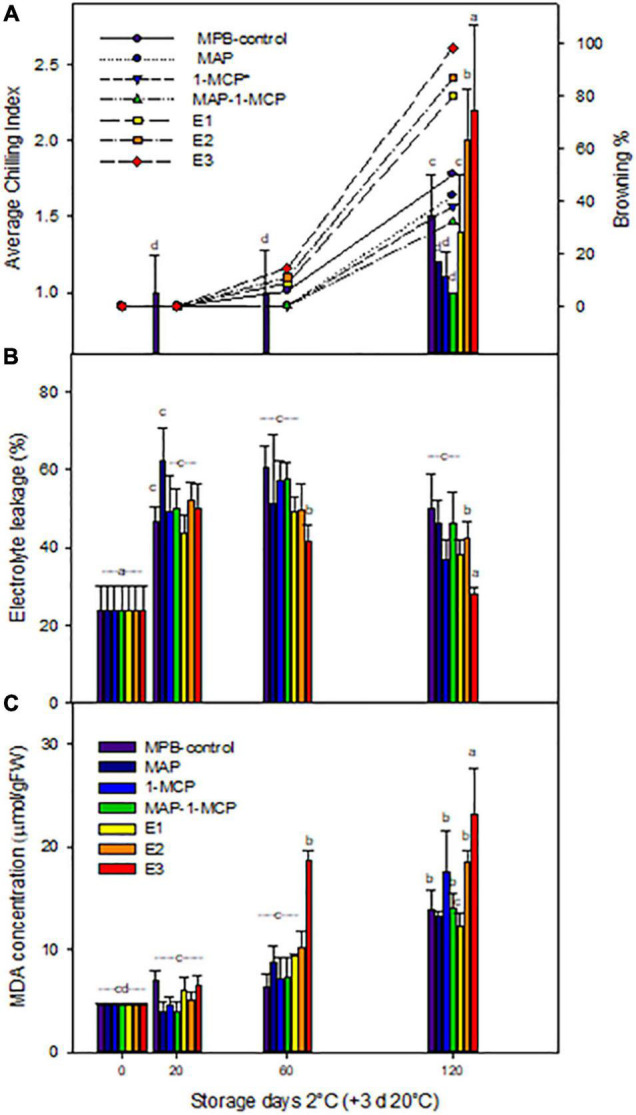
Chilling injury on ‘Wonderful’ pomegranate fruits. Pomegranates were stored at 2°C for up to 120 days and rewarmed for an additional 3 days at 20°C. Average chilling index and browning percent **(A)** and effects on electrolyte leakage percent **(B)** and lipid peroxidation (MDA concentration, mmol/gFW^–1^) **(C)** on the peel. The fruit was stored in macroperforated bags like control treatment (MPB-control), under a modified atmosphere (MAP), treated with ethylene inhibitor (1-MCP), (1 μg L^–1^), under combined treatment (MAP/1-MCP), and treated with increasing exogenous ethylene application (E1, E2, and E3; 0.5–1.5 μg L^–1^). Samples were taken at 0, 20, 60, and 120 days. In addition, a group maintained at room temperature (20°C) was used as a control (C). The results of chemical analysis are presented as the mean values ± SE considering five independent extractions and five technical replicates. Values followed by a different lowercase case letter are significantly different (*p* < 0.05).

The chilling injury in pomegranate is characterized by the browning of the peel surface. In this study, the CI appeared in ‘Wonderful’ pomegranates at 120 days of cold storage (3 days at 20°C), and it increased rapidly throughout the remaining shelf life. 1-MCP treatment delayed and reduced the CI index of pomegranate storage at cold temperatures (Figure 1). The average CI was 79.87 and 62.32% lower in 1-MCP-treated fruit than in the control fruit on the 120th and 160th days of storage, respectively.

Our previous studies showed that scalding on the blossom end of pomegranates was observed in MPB-control-stored fruits starting at 20 days and in E1-, E2-, and E3-treated fruits at 60 days of storage (Valdenegro et al., 2018), with an increase in the severity of chilling (>50%) from 100 days of cold storage, potentially related to senescence, decay, and deterioration (Defilippi et al., 2006). However, in this study, MAP and MAP/1-MCP treatments showed a CI symptom severity of 50% at 120 days of cold storage, indicating a delay of CI symptoms. Our previous studies showed an increase in ethylene production in E2- and E3-treated fruits at 20 days (Valdenegro et al., 2018), without a correlation between CO_2_ levels and visual symptoms during cold storage of pomegranate.

The modified atmosphere, e.g., the use of Xtend™ films, has been suggested as a good alternative to reduce dehydration, humidity, decay, and CI, preserving the hardness and shriveling of pomegranate (Porat et al., 2009; Valdenegro et al., 2018) and other fruits (Pesis et al., 2000; Aharoni et al., 2008), compared with another bag packaging. In this study, the average CI index (ACII) was observed from 20 days in MPB-control fruits and was highest in E2 and E3 at 120 days (+3 days at 20°C) (Figure 1). The severity of CI symptoms (Figure 1) increased on MPB-control fruits, E2 (11%), and E3 (14%) fruit from 60 days at 2°C (+3 days at 20°C), with a severity major of 40% from 120 days for all treatments, being higher for MPB-control and E3 (greater than 100%). In contrast, under the 1-MCP and MAP/1-MCP treatments, high CI symptom severity tended to show only after 120 days. In cherry fruits, the application of 1-MCP (0.3 and 1 ml L^–1^) reduced decay, pitting rot development, and respiration depending on cultivar during cold storage (Caleb et al., 2012; Piazzolla et al., 2015; Li et al., 2016). Contrary to 1-MCP treatment, it has been reported that controlled atmosphere storage conditions meaningfully reduced the incidence and severity of scald in pomegranate storage at 7°C (Defilippi et al., 2006). However, the delay of CI symptoms in pomegranate at temperatures below 5°C for 60 days can be associated with ethylene signaling (Valdenegro et al., 2018). Concerning the quality parameters, we previously showed that the peel color, TSS, and acidity of pomegranate arils were affected only by cold storage, with no significant differences between treatments, similar to Artés et al. (2000) and Casares et al. (2019).

### Relations Between Membrane Integrity and Ethylene Application During Cold Storage

Loss of membrane permeability is the first effect of cold damage in cold-sensitive species (McCollum and McDonald, 1991; Sala et al., 1998; Ben-Amor et al., 1999). In non-climacteric fruits such as grapes, electrolyte leakage is not previous to CI symptoms (Bellincontro et al., 2006). In the same way, this study showed an increase in the appearance of visual symptoms of CI parallel to electrolyte leakage in pomegranate peel (Casares et al., 2019). 1-MCP alone and MAP/1-MCP combination treatments decreased significant membrane damage compared to MBP and ethylene treatment at 20 days (Figure 1C), suggesting solute leakage, oxidative damage, and chilling membrane damage in pomegranate peel as a function of exogenous ethylene dose and O_2_ levels during the first days of storage, as reported by Valdenegro et al. (2018).

### Changes in Fatty Acids in the Peel

**FIGURE 2:**
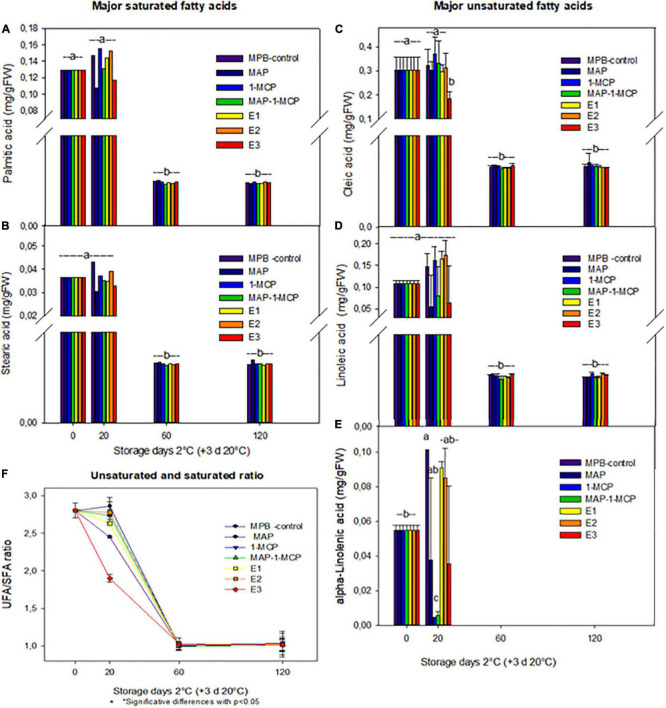
Fatty acids in the peel of ‘Wonderful’ pomegranate during cold storage. Effects of low-temperature storage at 2°C and rewarmed for an additional 3 days at 20°C on the content of principal fatty acids (mg/g FW): palmitic acid **(A)**, stearic acid **(B)**, oleic acid **(C)**, linoleic acid **(D)**, and alpha-linolenic acid **(E)** in the peel. Unsaturated and saturated ratio **(F)**. The fruit was stored in macroperforated bags like MPB-control, under a MAP, treated with 1-MCP, under combined treatment (PMA/1-MCP) and treated with increasing exogenous ethylene applications (E1, E2, and E3). Samples were taken at 0, 20, 60, and 120 days. The results of chemical analysis are presented as the mean values ± SE considering five independent extractions and five technical replicates. Values followed by a different lowercase case letter are significantly different (*p* < 0.05).

The decompartmentalization and ion leakage are products of changes in lipid constituents during damage to membrane structures (Casares et al., 2019). The decrease in unSFA content might affect the phase transition of membrane lipids. In loquat fruit, high levels of CI have been associated with a decrease in lipid unsaturation (Cao et al., 2011). Additionally, in pear fruit, some authors showed that preserved unsaturated lipid content and membrane fluidity enhance tolerance to chilling stress (Zheng et al., 2008; Cao et al., 2011; Jin et al., 2014). In this study, five major fatty acids (FAs) of membrane lipids were identified, including three unsaturated FAs, linoleic acid (18:2), linolenic acid (18:3), and alpha-linolenic acid (18:3), and two saturated FAs, palmitic acid (16:0) and stearic acid (18:0). Other authors found that linoleic acid, palmitic acid, and oleic acid are present in the peel and aril juice (Bar-Ya’akov et al., 2019). Additionally, the relative composition of individual FA components (Figure 2) and the unsaturated/saturated FA ratio (uns/sat FA ratio) were calculated to provide further insights into FA metabolism patterns under cold storage (Figure 2F). From 60 days, the contents of fatty acids were reduced during all treatments (Figure 2). However, when analyzing the unSFA and saturated acid rates, treatments with 1-MCP showed higher values. The most abundant fatty acids, such as linoleic acid and linolenic acid, and the ratio of unSFA to SFAs were higher in 1-MCP treatments (only and combined with MAP) than in MPB-control-treated fruits (Figure 2). These results suggest that 1-MCP might help to maintain the normal function of membranes and reduce CI in pomegranate fruit during cold storage. Our results may be aligned with Cheng et al. (2015), who previously established a relief mechanism of CI by regulating the metabolism of fatty acids and energy in pears. A high ratio of unSFA to SFAs in cell membranes could prevent leakage and MDA content increase, thereby preventing membrane peroxidation and damage. In addition, the change in membrane unsaturation was reflected by the alteration of the unsaturated/saturated FA ratio. During the first 20 days, a significantly higher unsaturated/saturated FA ratio was found in all 1-MCP treatments than in MPB-control fruits. Previous studies have shown that the cell membranes adapt, changing their lipid composition and ion channel transport and receptor protein activity by the effect of environmental temperature changes (Welti et al., 2002; Holthuis and Menon, 2014; Bustamante et al., 2018; Baghel et al., 2021).

### Enzymatic Activities Related to Browning in ‘Wonderful’ Pomegranate Fruits: Phenylalanine Ammonia-Lyase and Polyphenol Oxidase Activities

**FIGURE 3:**
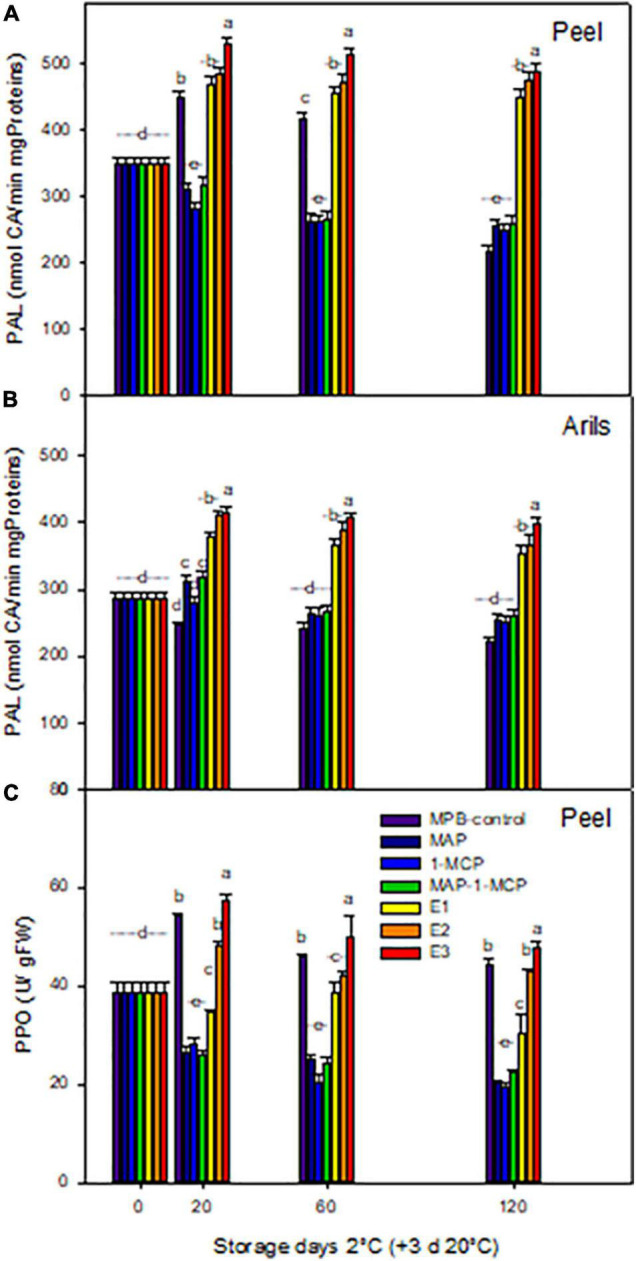
Enzymatic activities related to browning in ‘Wonderful’ pomegranate fruits. Pomegranate was stored at 2°C for up to 120 days and 3 at 20°C. Effect of storage on phenylalanine ammonia lyase (PAL) activity (nmols of cinnamic acid/min × g proteins) in peel **(A)** and arils **(B)** and polyphenol oxidase (PPO) activity (units of activity/g FW) in the peel **(C)**. The fruit was stored in macroperforated bags like MPB-control, under a MAP, treated with 1-MCP, under combined treatment (PMA/1-MCP), and treated with increasing exogenous ethylene applications (E1, E2, and E3). Samples were taken at 0, 20, 60, and 120 days. The results of chemical analysis are presented as the mean values ± SE considering five independent extractions and five technical replicates. Values followed by a different lowercase case letter are significantly different (*p* < 0.05).

An increase in PAL activity is a cold-induced response that could be stimulated in fruit showing chilling damage, and that response may occur concomitantly with the development of chilling symptoms (Lafuente et al., 2003). The fruits of MAP, 1-MC, and combined treatments presented less PAL activity than MBP and ethylene treatment at 20 and 60 days in the peel (Figure 3A), while a significant increase of PAL was observed in all concentrations of ethrel treatment in the arils compared to other treatments (Figure 3B). Figure 3C shows the effect of the treatments and the storage time on PPO activity expressed as a variation in absorbance at 420 nm per min and gram of tissue. In the control treatment (MPB-control), the enzymatic activity values remained practically constant during the storage period tested, being higher in the first 20 days of evaluation. For the treatments with atmospheric modification or 1-MCP treatment, the activity was reduced by 50%, while a great increase in activity was observed from 20 days for all levels of exogenous ethylene treatments in a dose-dependent manner. The harvest time to chilling tolerance is very important for ‘Wonderful’ pomegranate, so full ripeness shows a greater capacity to withstand cold stress conditions (Kashash et al., 2016). At this stage, the enzymatic and non-enzymatic antioxidant systems have a greater capacity to delay the appearance of symptoms of damage caused by exposure to cold (Ben-Arie and Or, 1986; Marangoni et al., 1996; Matityahu et al., 2013; Jin et al., 2014). However, it is not enough to counteract stress, destabilizing cell membranes. In prolonged periods of exposure to cold, the modification of the surrounding atmosphere can offer protection by delaying and reducing the level of damage (Pareek et al., 2015; Li et al., 2016).

### Total Antioxidant Capacity and Antioxidant Molecules

**FIGURE 4:**
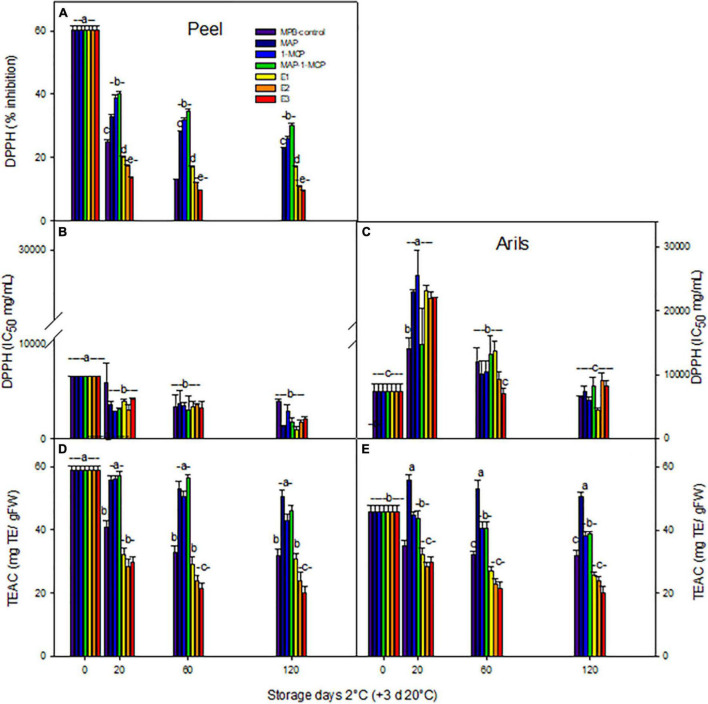
Total antioxidant capacity of ‘Wonderful’ pomegranate during cold storage. Effects of low-temperature storage at 2°C and rewarmed for an additional 3 days at 20°C on DPPH activity:% inhibition in peel **(A)**, IC_50_ (mg/ml) in peel **(B)**, arils **(C)**, TEAC (mgTE/gFW) in peel **(D)**, and arils **(E)** of pomegranate. The fruit was stored in macroperforated bags like MPB-control, under a MAP, treated with 1-MCP, under combined treatment (PMA/1-MCP), and treated with increasing exogenous ethylene applications (E1, E2, and E3). Samples were taken at 0, 20, 60, and 120 days. The results of chemical analysis are presented as the mean values ± SE considering five independent extractions and five technical replicates. TE: Trolox equivalent. Values followed by a different lowercase case letter are significantly different (*p* < 0.05).

The antioxidant activity by DPPH activity (Figures 4A–C) and TEAC (Figures 4D,E) decreased considerably during storage in all treatments in the peel. The IC_50_ value achieved significantly lower values in the peel (Figure 4B) than in arils (Figure 4C). In arils, an increased TEAC value product by exposure to cold stress was present, being higher in modified atmosphere and 1-MCP treatments (Figures 4D,E). However, the rate of decrease was lower in peel and arils treated with MAP or combined treatment MAP-1-MCP, compared to MPB-control treatment.

**FIGURE 5:**
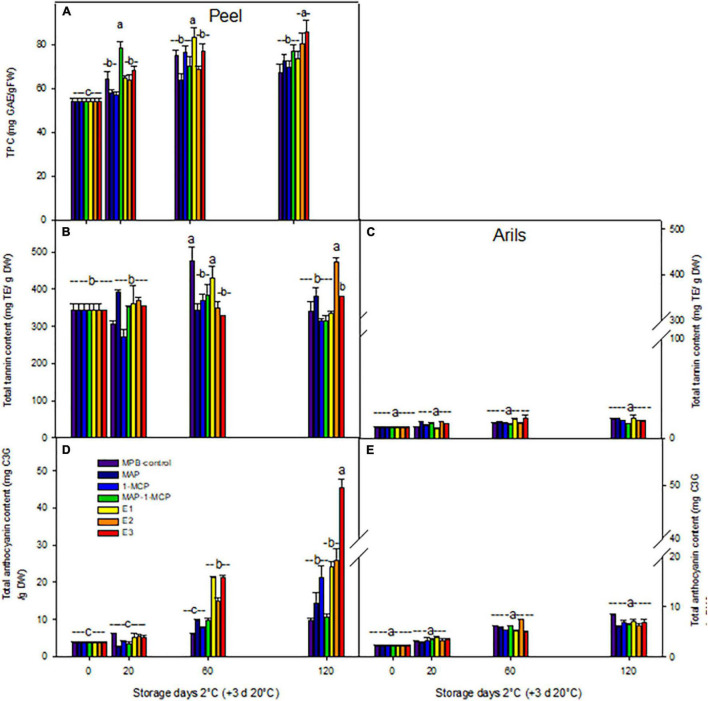
Effect of cold storage on the antioxidant molecules of ‘Wonderful’ pomegranate. Effects of low-temperature storage at 2°C and rewarmed for an additional 3 days at 20°C on total polyphenol content (TPC; mg GAE/gFW) in the peel **(A)**, total tannin content (mg TE/DW) in the peel **(B)**, arils **(C)**, total anthocyanin content [mg cyanidin-3-glucoside (C3G)/g DW] in the peel **(D)**, and arils **(E)** of pomegranate. The fruit was stored in macroperforated bags like MPB-control, under a MAP, treated with 1-MCP, under combined treatment (PMA/1-MCP), and treated with increasing exogenous ethylene application (E1, E2, and E3). Samples were taken at 0, 20, 60, and 120 days. The results of the chemical analysis are presented as the mean values ± SE considering five independent extractions and five technical replicates. Values followed by a different lowercase case letter are significantly different (*p* < 0.05).

The ethylene treatments showed higher browning in the peel, while the decrease in the total polyphenol content (TPC) in pomegranate peel (data not shown) may be due to the decomposition of TPC as a result of enzymatic activity during storage (Elfalleh et al., 2012); it could also be due to the oxidation or polymerization of TPC or the breakdown of the cell structure throughout product aging (Xie et al., 2019). One of the main causes of fruit browning is related to PPO activity, which reduces TPC. Increasing the activity of the PPO enzyme in browned peel causes a relative decrease in the phenol levels. Enzymatic oxidation of TPC, leading to browning of the peel in ethylene treatments, is probably conducted by the PPO enzyme, which is due to the highly reactive o-quinones forming brown colored polymers, leading to fruit browning (Shivashankar et al., 2012). In arils, the color is related to anthocyanin content, which contains cyanidin, pelargonidin, and delphinidin; color can be beneficial to health due to its antioxidant properties (Miguel et al., 2004; Naing and Kim, 2021). (Figures 5A–C) an increased concentration of anthocyanins in the peel (200, 5%) with a maximum value of 120 days was detected in ethylene treatments. However, its rate was lower in the 1-MCP or MAP-1-MCP treatments compared to the MPB-control treatment (Figure 5D). The lowest anthocyanin content was related to the MPB-control in arils and peel (Figures 5D,E). The tannin content remained stable over time in the peel in all treatments, without significant differences between treatments, slightly increasing its aril content from 20 days. It is important to note that the results presented in the current research are related to the ‘Wonderful’ cultivar, and the changes in tannins and anthocyanins could be different during cold temperatures in other varieties (Miguel et al., 2004; Alighourchi et al., 2008). Several authors point out the differences in varieties with respect to the abundance of anthocyanins in arils and skin (Zhao and Yuan, 2021). The most abundant arils are usually delphinidin 3,5-diglucoside, delphinidin 3-glucoside, cyanidin 3,5-diglucoside, cyanidin 3-glucoside, pelargonidin 3,5-diglucoside, and pelargonidin 3-glucoside (Alighourchi et al., 2008; Zhao et al., 2013). Zhao et al. (2013) studied Chinese varieties and found high variability in anthocyanins in the peel, presenting only pelargonidin and cyanidin derivatives. Naing and Kim (2021) point out that under cold stress conditions, reactive oxygen species are overproduced and cause oxidative damage to plants, and plants can produce anthocyanins, such as antioxidant responses, by neutralizing radicals with their hydroxyl groups to maintain normal cellular redox homeostasis.

**FIGURE 6:**
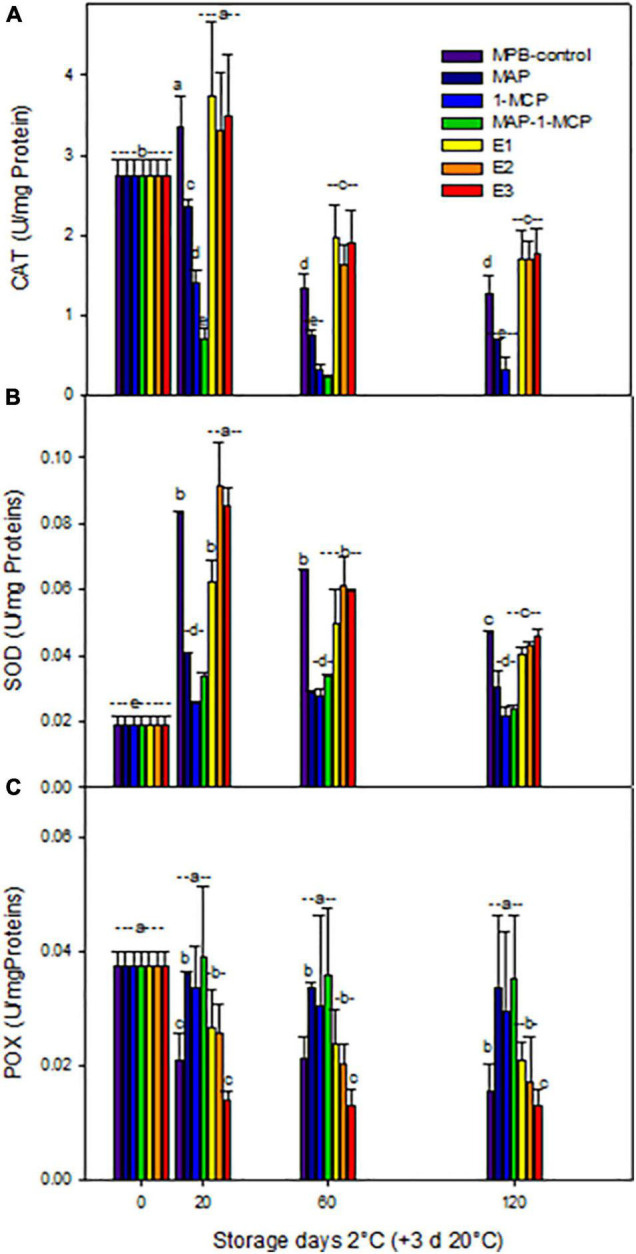
Antioxidant enzymatic response during cold storage of ‘Wonderful’ pomegranate. Effects of low-temperature storage at 2°C and rewarming for an additional 3 days at 20°C on the activities (U/mg Proteins) of catalase (CAT) **(A)**, superoxide dismutase (SOD) **(B)**, and guaiacol peroxidase (POX) **(C)** in the peel of a pomegranate. The fruit was stored in macroperforated bags like MPB-control, under a MAP, treated with 1-MCP, under combined treatment (PMA/1-MCP), and treated with increasing exogenous ethylene application (E1, E2, and E3). Samples were taken at 0, 20, 60, and 120 days. The results of the chemical analysis are presented as the mean values ± SE considering five independent extractions and five technical replicates. Values followed by a different lowercase case letter are significantly different (*p* < 0.05).

The modifications in the non-enzymatic antioxidant content of pomegranate fruits were related to the capacity of 1-MCP (alone or MAP combined) to increase their storability. With respect to enzymatic antioxidant systems, the CAT activity was increased in response to cold stress in ethylene treatments at 20 days at 2°C + 3 days 20°C, while MAP, 1-MCP, and combined treatment decreased activity by more than half of the ethrel treatments until the end of the assay (Figure 6A). The SOD activity (Figure 6B) had a similar trend with increased levels in ethylene treatments, while 1-MCP treatments (only and combined with MAP) remained stable. The POX activity (Figure 6C) showed the lowest levels in ethylene treatments. The lower browning of peel in pomegranate treated with 1-MCP can be due to higher antioxidant enzymes in the tissue, SOD, and CAT activities leading to a lower peel H_2_O_2_ accumulation and higher membrane integrity in agreement with lower electrolyte leakage and MDA accumulation observed in the treatment with major enzymatic antioxidant activity. These results are in concordance with Baghel et al. (2021), who studied the expression of these enzymatic activities. Additionally, the lower peel browning in pomegranate fruit of the combined MAP/1-MCP treatment resulted from a higher peel PAL/PPO enzyme activity ratio.

However, interestingly, the highest contents of anthocyanins and hydrolyzed tannins were detected when exogenous ethylene was applied to the fruits like a response to stress condition by cold temperature, with an increase in functional molecules. The treatment of exogenous ethylene E3 reduced TEAC antioxidant activity by 50% in the peel (Figure 4D) and 33% in arils (Figure 4E). In the peel, the refrigerated storage reduced the non-enzymatic antioxidant capacity, while in the arils, this parameter was increased, in accordance with the increase in the anthocyanin level. The treatments with the inhibitor of the action of ethylene 1-MCP had a clear effect on the maintenance of the antioxidant capacity, so it seems that ethylene is involved in the non-enzymatic mechanisms of detoxification in this fruit. 1-MCP and modified atmosphere packaging could preserve the functional quality of fruit tissues. Our results coincide with Naing and Kim (2021), since other non-climatic fruits showed increases in non-enzymatic antioxidant activity as a product of exposure to cold storage, which reduced CI symptoms.

### Sensory Aspects

**FIGURE 7:**
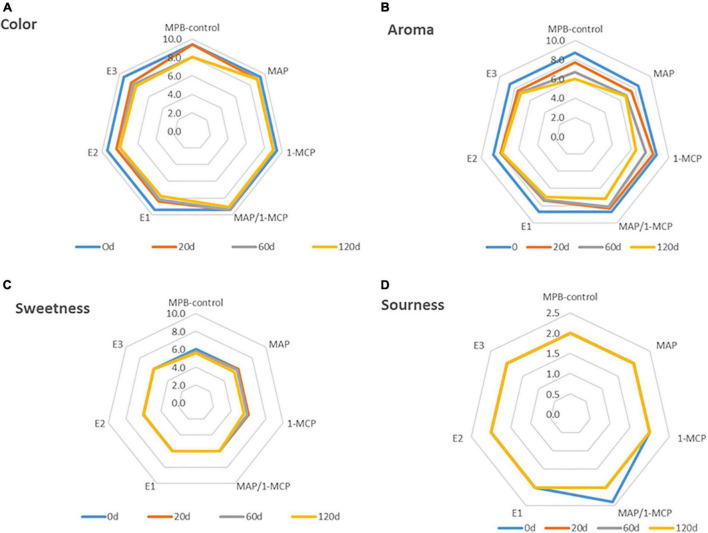
Sensory evaluation of ‘Wonderful’ pomegranate storage at low temperature. Effects of low-temperature storage at 2°C and rewarmed for an additional 3 days at 20°C on color, sweetness aroma, and sourness perception. The fruit was stored in macroperforated bags like MPB-control, under a MAP, treated with 1-MCP, under combined treatment (PMA/1-MCP), and treated with increasing exogenous ethylene application (E1, E2, and E3). Samples were taken at 0 **(A)**, 20 **(B)**, 60 **(C)**, and 120 days **(D)**. The evaluation was made using a hedonic scale of 10 points, with 4 as the minimum score for an acceptable attribute.

According to the evaluations of the panelists, few changes were perceived from 60 days (Figure 7). Less sweetness, color (luminosity), and aroma were recorded in MPB-control arils compared with other treatments (Figures 7C,D). Among the treatments used, the MAP and combined MAP-1-MCP had better sensory properties than the other treatments during cold storage. The MAP and the combined MAP-1-MCP treatment had no negative effect on the color, luminosity, or aroma of the arils. The ethylene exogenous treatments showed less color and luminosity and reduced appreciations of the aroma of arils. The results showed a significant acceptance of color and aroma for the storage pomegranate and a minor acceptance of sourness.

## Conclusion

This study found that during long-term cold storage of pomegranate fruits, the protective effect of MAP/1-MCP or MAP treatments delays the appearance of CI symptoms and reduces the oxidation of tissues. The antioxidant response was higher in these treatments. This long storage induces an important decrease in fatty acids, which is not reverted by any postharvest treatment. However, it was slowest in 1-MCP treatments. Furthermore, our results also showed that the metabolism of membrane lipid FAs was involved in the responses associated with CI. A higher unSFA/SFA ratio after 1-MCP treatments showed a protective effect in peel tissues. In addition, it is possible to increase the concentration of anthocyanins in the peel of cold-storage pomegranates treated with ethylene, similar to a tolerance mechanism to cold stress.

## Data Availability Statement

The raw data supporting the conclusions of this article will be made available by the authors, without undue reservation.

## Author Contributions

MV, MB, CH, LF, MS, LM, and IH contributed to the structure and focus of the manuscript. MV, MB, LF, MS, LM, IH, and RS performed the physical-chemical analysis. MV, MB, LF, and IH conducted an investigation. LF and MV contributed to the preparation, wrote the original draft of the manuscript, review and editing, supervision, and project administration. MV, LF, MS, LM, MB, CH, IF, and RS contributed to funding acquisition. All authors have agreed to the published version of the manuscript.

## Conflict of Interest

The authors declare that the research was conducted in the absence of any commercial or financial relationships that could be construed as a potential conflict of interest.

## Publisher’s Note

All claims expressed in this article are solely those of the authors and do not necessarily represent those of their affiliated organizations, or those of the publisher, the editors and the reviewers. Any product that may be evaluated in this article, or claim that may be made by its manufacturer, is not guaranteed or endorsed by the publisher.
